# HSV-1 ICP0 expression induces formation of nuclear TDP-43 assemblies

**DOI:** 10.1007/s13365-026-01349-z

**Published:** 2026-10-26

**Authors:** Shirley E. Braspenning, Adam T. Silhavy, Nora L. Herzog, Phoebe A. Chen, Angus C. Wilson, Ian J. Mohr

**Affiliations:** 1 Department of Microbiology, Tumor and Cell Biology, Karolinska Institutet, Stockholm, Sweden; 2 Department of Microbiology, New York University School of Medicine, New York, NY USA; 3 Institute for Systems Genetics, New York University Langone Health, New York, NY USA

**Keywords:** HSV-1, TDP-43, ICP0, Protein aggregation, Virus-host interactions

## Abstract

Toxic cytoplasmic aggregates containing the nuclear RNA-binding protein TDP-43 form in several neurodegenerative diseases; however, the molecular triggers driving aggregation remain unclear. Epidemiological evidence has linked latent and reactivating herpes simplex virus type-1 (HSV-1) infections to neurodegenerative disease onset, but it is unknown if HSV-1 infection impacts TDP-43 subcellular distribution. Here, we demonstrate that HSV-1 infection of epithelial cells and DRG-derived neurons results in nuclear TDP-43 redistribution and TDP-43 accumulation within distinct nuclear assemblies. Cytoplasmic TDP-43 was not detected under these conditions. Nuclear TDP-43 assembly occurred early in infection and was dependent upon expression of the critical ICP0 regulatory gene. Remarkably, ectopic ICP0 expression in uninfected cells was sufficient for nuclear TDP-43 assembly. Thus, expression of a single viral gene product encoded by a near ubiquitous human herpesvirus can alter TDP-43 nuclear localization. Moreover, our work identifies a potential mechanistic link between herpesvirus infection and neuronal disease factor mislocalization.

## Introduction

TAR DNA binding protein 43 (TDP-43) is an ubiquitously expressed RNA binding protein, with key roles in RNA localization, splicing and degradation (Tollervey et al. 2011; Cohen et al. 2011; Buratti and Baralle 2012). During homeostasis the protein is predominantly nuclear, with a smaller subset shuttling between the nucleus and cytoplasm (Ayala et al. 2008). Cytoplasmic aggregation and nuclear depletion of TDP-43 is seen as a hallmark of multiple neurodegenerative disorders including amyotrophic lateral sclerosis (ALS) and frontotemporal dementia (Neumann et al. 2006; Cairns et al. 2007; Taylor et al. 2016). Several post-translational modifications are thought to contribute to TDP-43 aggregation (Buratti 2018), with the presence of ubiquitinated and phosphorylated TDP-43 considered characteristic for proteotoxic aggregates (Neumann et al. 2006; Cairns et al. 2007; Hasegawa et al. 2008; Inukai et al. 2008). However, it is unclear how these aggregates form in vivo, and which molecular switches trigger aberrant nuclear or cytoplasmic localization. One hypothesis is that initial cellular stress leads to temporary accumulation of TDP-43 in the cytoplasm, creating a nuclear loss-of-function and a cytoplasmic gain-of-function phenotype that then drives further aggregation (Sun and Chakrabartty 2017; Hergesheimer et al. 2019). Other studies have suggested that proteotoxic cytoplasmic aggregation is preceded by nuclear condensates that form through liquid-liquid phase separation in response to reduced RNA-binding (Pérez-Berlanga et al. 2023; Scherer et al. 2024). Notably, multiple cellular stressors, including innate immune activation, induce the formation of RNA-depleted nuclear TDP-43 assemblies which are associated with impaired splicing of neuronal target genes (Huang et al. 2024). Although several mutations in TDP-43 and associated factors are known to accelerate disease progression upon initial accumulation, the potential involvement of extrinsic stressors, including virus infection, is understudied.

Herpes simplex virus type-1 (HSV-1) is an α-herpesvirus and neurotropic pathogen that establishes life-long latency in sensory neurons of the trigeminal and dorsal root ganglia. From here, the virus intermittently reactivates to cause cold sores, ocular infections, or in rare cases encephalitis. Given their ubiquitous prevalence and their propensity to establish latency in sites with direct connection to the central nervous system, α-herpesviruses have increasingly been linked to neurodegenerative disorders such as Alzheimer’s disease. Indeed, vaccination with either the live-attenuated or the subunit vaccine against varicella-zoster virus (VZV) has been shown to delay the onset of dementia significantly (Taquet et al. 2024; Eyting et al. 2025), and studies have suggested a correlation between HSV-1 reactivation and neurodegenerative disease (De Chiara et al. 2019; Dutton et al. 2025). However, it remains unclear how HSV-1 or VZV infection mechanistically alters neuronal health and homeostasis to contribute to disease. We have previously shown that TDP-43 promotes efficient HSV-1 replication in DRG-derived neuronal cells, but not epithelial cells or fibroblasts (Braspenning et al. 2025), suggesting a neuron-specific role for TDP-43 in HSV-1 infection. Whether the interaction between HSV-1 and TDP-43 is reciprocal, with the virus altering TDP-43 subcellular distribution and aggregation that have been linked with pathological outcomes, remains to be determined.

In this study, we investigated how HSV-1 infection affects the intracellular localization and subcellular distribution of TDP-43. Productive HSV-1 infection in epithelial cells leads to the formation and accumulation of intranuclear TDP-43 assemblies early in the virus productive growth cycle. Next, we show that these nuclear assemblies are distinct from other virus-induced nuclear domains and stress-induced nuclear TDP-43 bodies. Significantly, we identified a single viral protein, ICP0, as the driving factor for TDP-43 assembly. Finally, we extended our work to a more disease-relevant cell type, recapitulating our results in DRG-derived neuron-like cells.

## Results

### HSV-1 infection leads to the formation of intranuclear TDP-43 assemblies

**Fig. 1:**
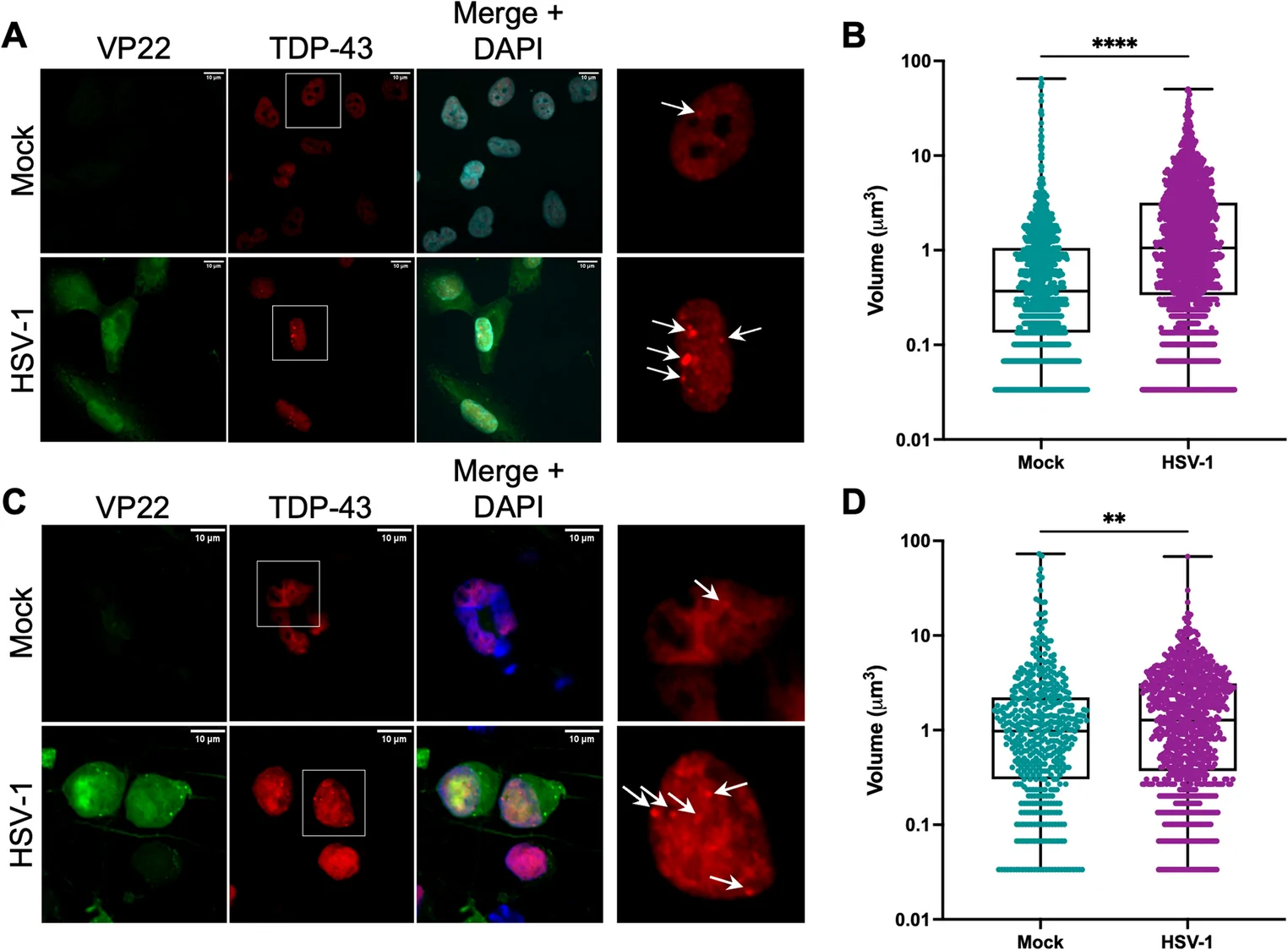
TDP-43 forms nuclear assemblies in HSV-1 infected cells. **A**) Immunofluorescence of ARPE-19 cells infected with HSV-1 KOS for 9 hours and probed for HSV-1 VP22 (green) and TDP-43 (red) and counterstained with DAPI (cyan) showing nuclear assemblies in infected cells (white box, zoomed in). **B**) Volume of individual TDP-43 assemblies in uninfected and HSV-1 KOS infected cells at 9 hpi. n=3 independent experiments, ≥18 cells per condition, ≥1201 assemblies, **** p < 0.0001 by lognormal Welch’s t-test. **C**) Immunofluorescence of differentiated HD10.6 cells infected with HSV-1 KOS for 24 hours and probed for HSV-1 VP22 and TDP-43 showing nuclear assemblies in Mock-infected and HSV-1 infected cells (white arrows, zoomed in). **D**) Volume of individual TDP-43 assemblies in Mock-infected and HSV-1 infected differentiated HD10.6 cells at 24hpi. n=3 independent experiments, ≥47 cells per condition, ≥485 assemblies ** p < 0.01 by lognormal Welch’s t-test.

To investigate whether TDP-43 localization is affected during HSV-1 infection, we infected human retinal pigmented epithelial cells (ARPE-19) with HSV-1 KOS for 9 h and performed indirect immunofluorescence to detect cellular TDP-43 and the viral protein VP22 as a marker of productive infection. In uninfected epithelial cells, TDP-43 was detected diffusely throughout the nucleus with nucleolar sparing (Fig. 1a, top panels). However, in HSV-1 infected cells, we observed the formation of nuclear TDP-43 assemblies (Fig. 1a, lower panels). Although TDP-43 is known to form small dynamic nuclear inclusions under homeostatic conditions (Pérez-Berlanga et al. 2023; Scherer et al. 2024), assemblies observed upon HSV-1 infection appeared to be larger and more numerous compared to those in uninfected cells. To determine the size of the TDP-43 assemblies, we took z-stack images with 0.5-µm step sizes and used these to estimate the volumes of individual assemblies. Indeed, HSV-1 infection significantly increased the volume of intranuclear TDP-43 assemblies compared to uninfected (Mock) cells [geometric mean volume of 0.9371 µm^3^ vs. 0.3715 µm^3^ respectively] (Fig. 1b). Notably, we were unable to detect any cytoplasmic relocalization or assembly of TDP-43 during lytic HSV-1 infection.

### TDP-43 forms intranuclear inclusions in HSV-1 infected neurons

Our previous work established that TDP-43 is uniquely required for efficient HSV-1 replication in differentiated HD10.6 cells (Braspenning et al. 2025). These cells are originally derived from dorsal root ganglia neurons and can quickly be differentiated into functional neuron-like cells (Raymon et al. 1999; Dochnal et al. 2026). As TDP-43 localization is also key to neuronal homeostasis, we analyzed the subcellular localization of TDP-43 in differentiated HD10.6 cells infected with HSV-1. In HSV-1 infected HD10.6 cells (Fig. 1c), significantly larger intranuclear TDP-43 assemblies were observed compared to Mock [geometric mean volume of 1.021 µm^3^ vs. 0.8125 µm^3^ respectively] (Fig. 1d) at 24 hpi but the effect size was smaller compared to epithelial cells. Together, our data demonstrate that HSV-1 infection induces intranuclear TDP-43 assembly in neuron-like HD10.6 cells, establishing this phenotype exists across multiple cell types, including more disease-relevant cellular models.

### GFP-TDP43 recapitulates HSV-1 induced nuclear assembly

**Fig. 2:**
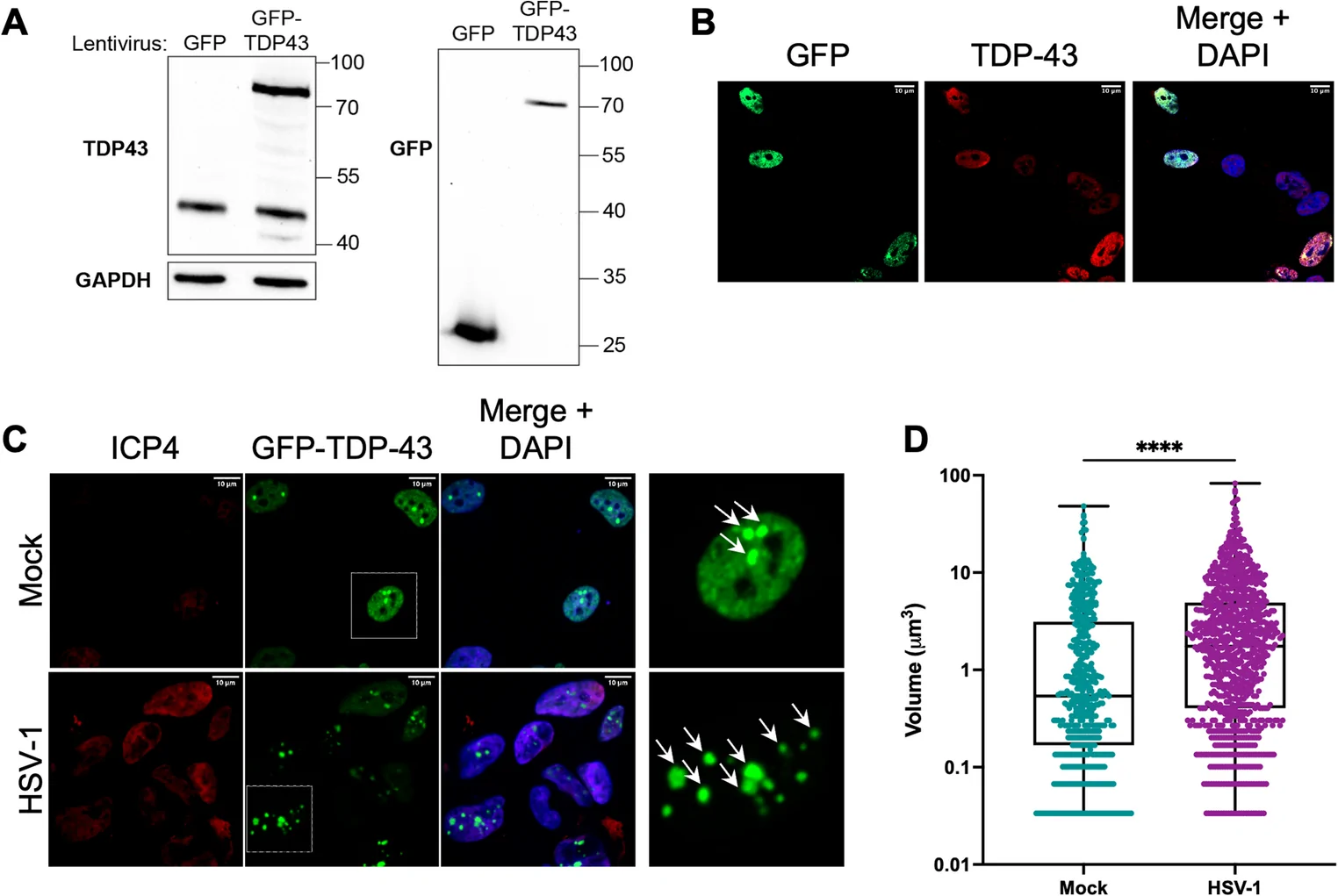
Validation of GFP-TDP43 as a reporter of HSV-1-induced nuclear assembly. **A**) Immunoblots for TDP-43 and GFP confirming expression of GFP-tagged TDP-43 in ARPE-19 cells at 3 days post-transduction with either a GFP-expressing lentivirus or GFP-TDP43 expressing lentivirus. GAPDH was used as a loading control. **B**) Immunofluorescence for TDP-43 and GFP in GFP-TDP43 transduced ARPE-19 cells **C**) Immunofluorescence for GFP (green) and HSV-1 ICP4 (red) in GFP-TDP43 transduced ARPE-19 cells infected with HSV-1 KOS for 9 hours. **D**) Volume of individual GFP-TDP43 assemblies at 9 hpi. n=3 independent experiments, ≥38 cells per condition, ≥492 assemblies **** p < 0.0001 by lognormal Welch’s t-test

In order to increase the sensitivity of TDP-43 detection and exclude nuclear assembly as an antibody-dependent artefact, we transduced ARPE-19 cells with a lentivirus expressing TDP-43 fused to GFP at the N-terminus (Schmidt et al. 2019) to facilitate direct observation of TDP-43 localization. Ectopic expression of GFP-TDP43 was validated by immunoblot for TDP-43 and GFP (Fig. 2a), showing abundant detection of both endogenous and GFP-tagged exogenous TDP-43 without apparent changes to endogenous TDP-43 levels. Overexpression of TDP-43 was further confirmed by immunofluorescence, showing enhanced detection of TDP-43 in GFP+ cells (Fig. 2b). Consistent with our observation of endogenous TDP-43, GFP-TDP43 was diffusely nuclear in uninfected cells, and HSV-1 infection led to formation of nuclear assemblies of GFP-TDP43 (Fig. 2c). Again, the volume of GFP-TDP43 assemblies upon HSV-1 infection was significantly increased compared to Mock [geometric mean volume of 1.416 µm^3^ vs. 0.6486 µm^3^ respectively] (Fig. 2d). Although GFP-TDP43 assemblies were larger than endogenous TDP-43 assemblies, the relative increase in size upon HSV-1 infection was similar [2.18-fold vs. 2.5-fold respectively], suggesting that in our system overexpression of TDP-43 does not enhance assembly formation.

### Intranuclear TDP-43 assemblies are not enriched for HSP70 proteins

**Fig. 3:**
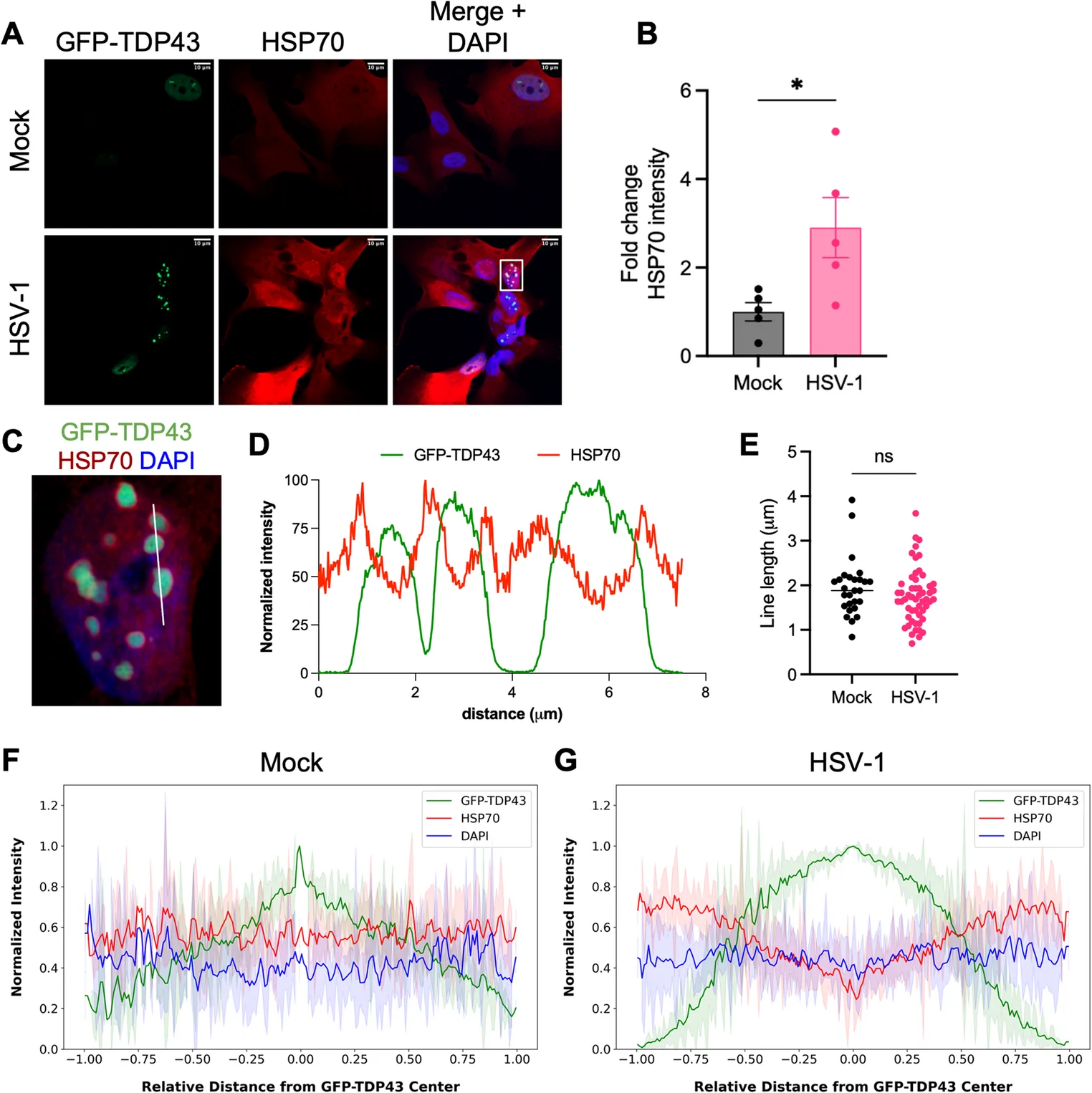
HSV-1 induced nuclear TDP-43 assemblies are not enriched for the HSP70 chaperone protein**.** (**A**) Immunofluorescence for GFP (green) and HSP70 (red) in Mock-infected or HSV-1 infected GFP-TDP43 transduced ARPE-19 cells at 9 hpi. (**B**) Fold change of HSP70 fluorescence intensity in HSV-1 infected cells normalized to average fluorescent intensity of Mock-infected cells with the same acquisition settings. * *p* < 0.05 by Welch’s t-test. (**C**) Enlarged area of HSP70 with a line drawn through three GFP-TDP43 assemblies with (**D**) Normalized fluorescence intensity of GFP-TDP43 and HSP70 along the line. (**E**) Real line lengths drawn through individual GFP-TDP43 assemblies. ns = non-significant by Welch’s t-test. F, G) Moving average (solid line) of DAPI, GFP-TDP43 and HSP70 normalized intensities through *n* = 27 (Mock) and *n* = 54 (HSV-1) individual GFP-TDP43 assemblies with 95% confidence intervals (shaded area) for Mock-infected (**F**) and HSV-1 (**G**) infected cells

Next, we investigated if the TDP-43 nuclear assemblies resemble virus- or stressed induced nuclear structures. Of particular interest were anisosomes, nuclear structures in which TDP-43 surrounds a core of molecular chaperone proteins such as HSP70 (Yu et al. 2021). Thus, we investigated whether HSV-1 induced TDP-43 assemblies also contain HSP70. Immunofluorescence for HSP70 revealed diffuse cytoplasmic staining in uninfected cells whereas HSV-1 infection induced HSP70 nuclear localization (Fig. 3a) and significantly increased signal intensity (Fig. 3b). In some cells, but not all, HSP70 was concentrated in subnuclear structures (Fig. 3c). To determine localization of HSP70 in relation to GFP-TDP43 assemblies more precisely, we first plotted fluorescent signal intensity along a line crossing three GFP-TDP43 assemblies (Fig. 3c-d). These profiles showed that HSP70 signal peaks at the periphery of the GFP-TDP43 assemblies and is essentially absent within. To validate this observation, we generated similar intensity profiles across 54 GFP-TDP43 assemblies in HSV-1 infected cells and 27 in Mock-infected cells, using lines of similar lengths (Fig. 3e). To enable comparison of these assemblies, the center of the assembly was defined by the peak of GFP-TDP43 signal and distances were normalized relative to the center of the assembly. We next calculated the moving average of GFP-TDP43, HSP70 and DAPI signal along the normalized distance axis, and plotted these values (solid lines) along with a 95% confidence interval (shaded area) (Fig. 3f-g). In accordance with our earlier data, GFP-TDP43 assemblies were larger in HSV-1 infected cells, as evidenced by a broader GFP-TDP43 signal peak in HSV-1 infected cells compared to Mock-infected controls. Notably, whereas HSP70 signal remained relatively constant across GFP-TDP43 assemblies in Mock-infected cells (Fig. 3f), the signal was markedly lower within HSV-1 induced assemblies compared to the surrounding nuclear region (Fig. 3g). Together, these data showed that HSV-1 induced GFP-TDP43 assemblies are not enriched for HSP70 proteins, suggesting that these assemblies are distinct structures from anisosomes.

### Formation of intranuclear TDP-43 assemblies is dependent on viral protein synthesis and ICP0 expression

**Fig. 4:**
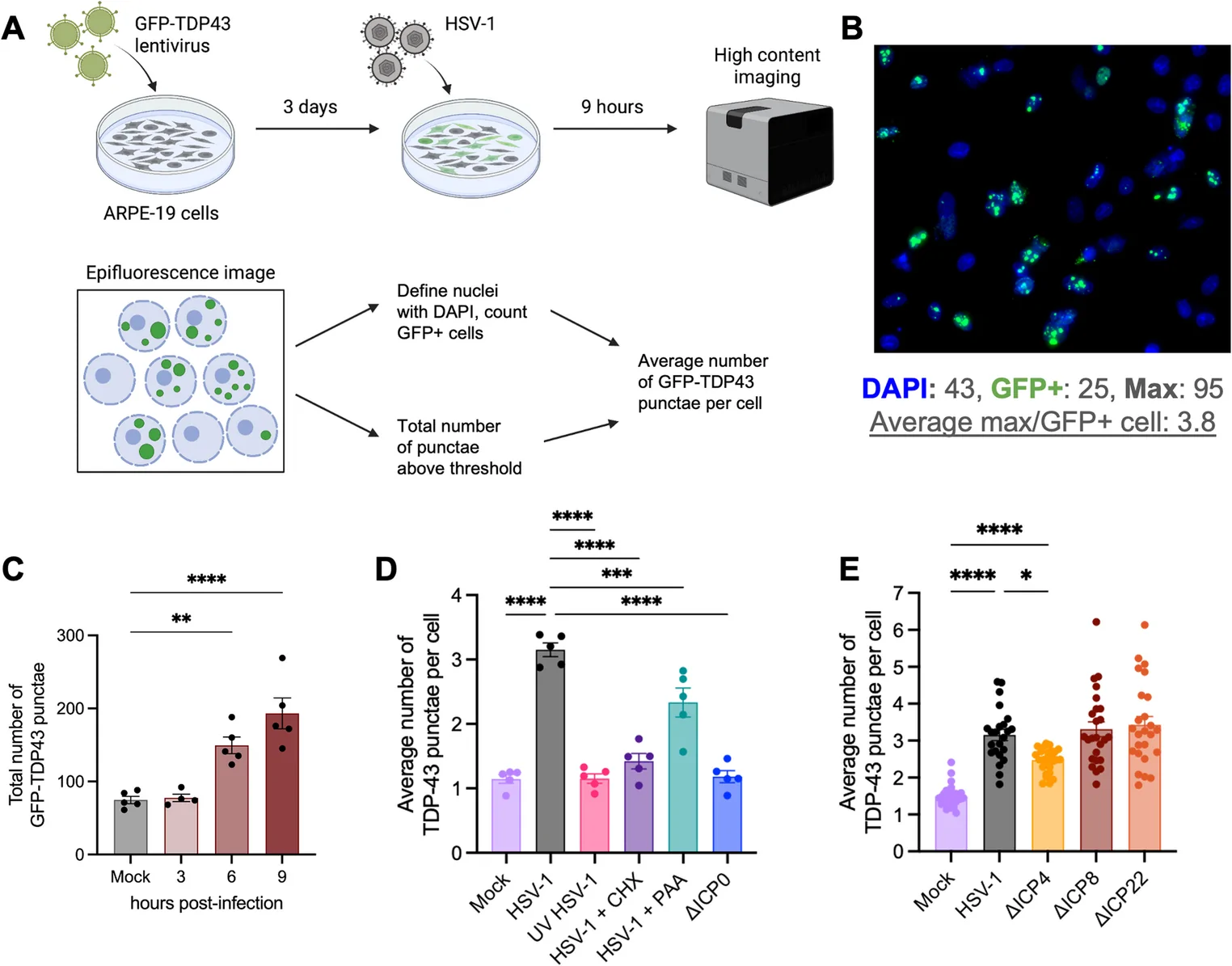
Formation of nuclear TDP-43 assemblies is dependent on viral protein production but not replication. **A**) Experimental layout for high-content imaging. ARPE-19 cells were transduced with GFP-TDP43 lentivirus for 3 days, and infected with HSV-1 at MOI=3 for 9 hours. Using a high-content imager, epifluorescent images were obtained for DAPI and GFP. From these images the number of GFP+ cells was determined, as well as the total number of assemblies per image to calculate the average number of TDP-43 assemblies per cell. **B**) Representative image of GFP-TDP43 transduced cells infected with HSV-1, showing DAPI and GFP-TDP43 signal with corresponding cell and maximum count. **C**) GFP-TDP43 assemblies per imaging field in Mock-infected and in HSV-1 KOS infected cells at 3, 6 and 9 hpi. **D**) Average number of GFP-TDP43 assemblies per cell infected for 9 hours with HSV-1 KOS, UV-inactivated HSV-1 KOS or HSV-1 KOS with cycloheximide (CHX) or phosphonoacetic acid (PAA) or HSV-1 ΔICP0. **E**) Average number of GFP-TDP43 assemblies per cell upon infection with HSV-1 KOS, HSV-1 ΔICP4, ΔICP8 or ΔICP22. * p<0.05, *** p< 0.001, and **** p<0.0001 by One-Way ANOVA with Dunnett’s multiple comparisons test.

We next sought to identify which viral factors are responsible for TDP-43 assembly. To determine at which phase of HSV-1 infection intranuclear TDP-43 assemblies formed, we developed a high-content imaging pipeline to detect and count larger, high fluorescent intensity GFP-TDP43 foci and determine the average number of foci per cell (Fig. 4a-b). This approach allows for fast screening of several conditions in a high-throughput set-up. First, we infected GFP-TDP43 transduced cells, and imaged cells at 3, 6 and 9 h post-infection (hpi), as well as uninfected cells (Mock). Using this approach, we detected similar numbers of GFP-TDP43 assemblies in Mock-infected cells and at 3 hpi, whereas total GFP-TDP43 assemblies were significantly increased at 6 and 9 hpi (Fig. 4c). To delineate in which kinetic stage, *immediate-early*,* early* or *late*, GFP-TDP43 assemblies form, we first infected cells with an UV-inactivated virus or in the presence of cycloheximide (CHX). In these conditions, we observed no increase in average number of GFP-TDP43 nuclear assemblies per cell compared to uninfected cells (Mock), meaning that both viral gene expression and *de novo* protein synthesis are required for the formation of those assemblies (Fig. 4d). Surprisingly, although TDP-43 assemblies increased late in infection (Fig. 4c), inhibition of viral DNA replication with phosphonoacetic acid (PAA) did not fully prevent TDP-43 assembly formation (Fig. 4d). Thus, TDP-43 assembly during HSV-1 infection is dependent on either *immediate-early* or *early* viral proteins. In the same experiment, we also assessed the ability of a virus deficient for the *immediate-early* protein ICP0 (ΔICP0, HSV-1 mutant 7134) to induce GFP-TDP43 assemblies. Interestingly, the average number of assemblies was similar to Mock-infected cells, suggesting that ICP0 might have a role in the formation of GFP-TDP43 assemblies.

To determine if other viral proteins could also be involved in nuclear TDP-43 assembly, we tested a subset of HSV-1 viruses with mutations known to alter nuclear remodeling for their ability to induce nuclear GFP-TDP-43 assembly (Fig. 4e). In accordance with the chemical inhibition of DNA replication by PAA, genetic inhibition using a virus mutant for the *early* single-strand DNA-binding protein ICP8 (ΔICP8, HSV-1 mutant d301) still showed TDP-43 assembly formation equivalent to wild-type KOS. As we have previously shown that ICP4 expression is sufficient to remodel nuclear architecture and drive accumulation of artificial nuclear condensates (Herzog et al. 2026), we tested a virus mutant for ICP4 (ΔICP4, HSV-1 mutant n12) for its ability to induce TDP-43 assemblies. Although we observed a slightly reduced number of TDP-43 assemblies per cell compared to HSV-1 KOS, the average number of assemblies still increased significantly in comparison to uninfected cells. Another *immediate-early* protein, ICP22, induces the formation of Virus-Induced Chaperone-Enriched (VICE) domains that contain misfolded proteins, proteasome components, and chaperone proteins (Burch and Weller 2004; Livingston et al. 2009; Metzger et al. 2025). To exclude the possibility that the observed TDP-43 nuclear assemblies were VICE domains, we included a virus mutant for ICP22 (ΔICP22, HSV-1 d22LacZ) and observed a similar average number of assemblies per cell compared to HSV-1 KOS. These results suggest that intranuclear assembly of TDP-43 during HSV-1 infection does not require ICP8 or ICP22 but might be partially dependent on ICP4 expression. It is noteworthy that ICP4 expression promotes nuclear localization of ICP0 during infection (Zhu et al. 1994), and thus the observed mild decrease in nuclear assemblies might be due to reduced availability of nuclear ICP0.

**Fig. 5:**
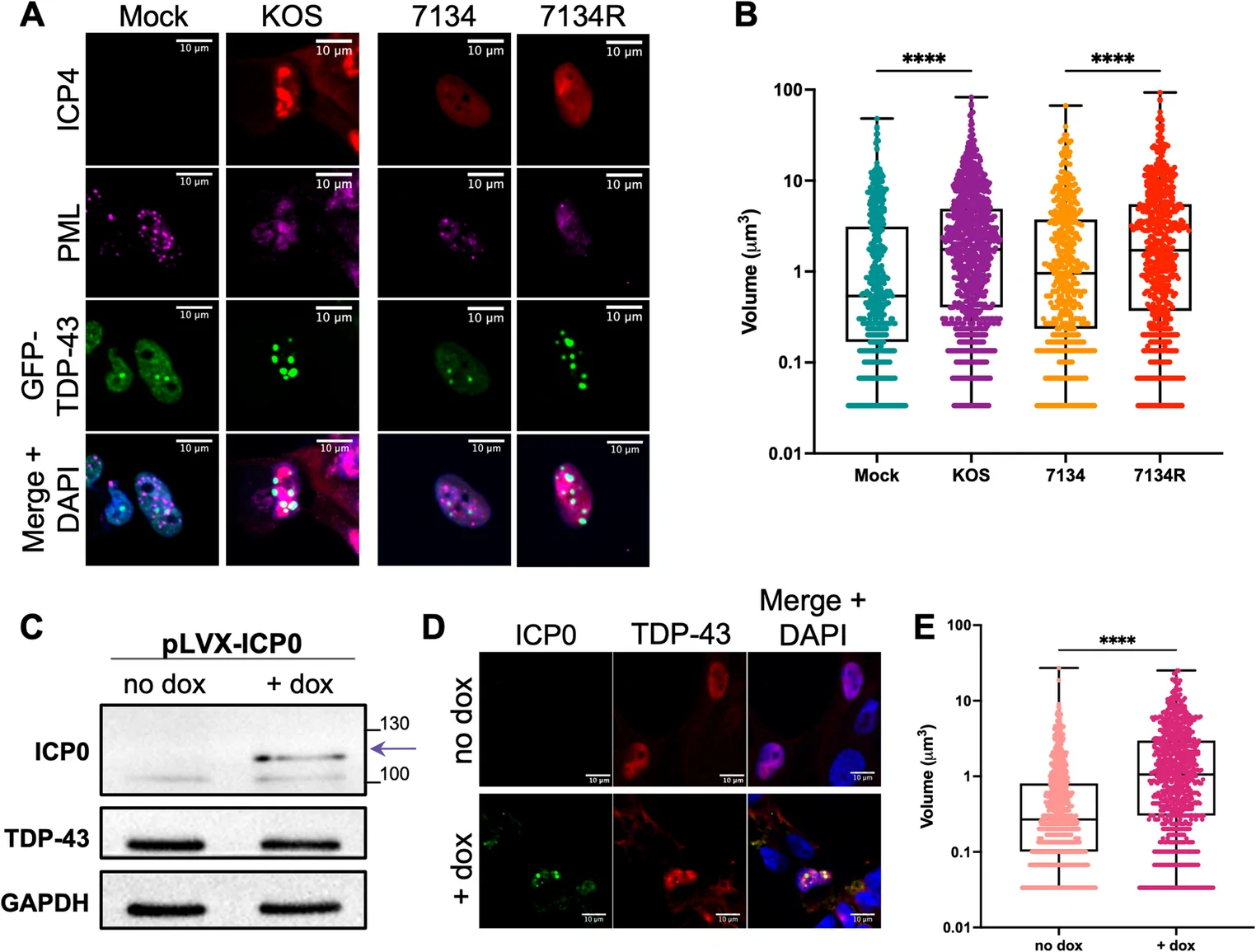
ICP0 expression is necessary and sufficient for the formation of nuclear TDP-43 assemblies. (A-B) ARPE-19 cells were transduced with GFP-TDP43 lentivirus for 3 days and infected with HSV-1 KOS, 7134 (ΔICP0) and 7134R for 9 hpi. (**A**) Immunofluorescence for HSV-1 ICP4 (red), PML (magenta) and GFP-TDP43 (green) and (**B**) Volume of individual GFP-TDP43 assemblies in 7134- and 7134R-infected cells were analyzed in addition to Mock- and KOS-infected cells from Fig. 2d. *n* = 3 independent experiments, ≥36 cells per condition, ≥462 assemblies **** *p* < 0.0001 by Brown-Forsythe ANOVA test with Holm-Šídák’s multiple comparisons test. (C-E) ARPE-19 cells were transduced with pLVX-ICP0 and treated with doxycycline for 9 h. (**C**) Immunoblot confirming the expression of ICP0 upon treatment with doxycycline (+ dox) as well as the abundance of TDP-43. The arrow indicates the top band corresponding to ICP0, whereas the lower band is non-specific. GAPDH was used as a loading control. (**D**) Immunofluorescence for ICP0 (green) and endogenous TDP-43 (red), counterstained with DAPI (blue) with (**E**) Volume of individual endogenous TDP-43 assemblies. *n* = 3 independent experiments, ≥16 cells per condition, ≥803 assemblies **** *p* < 0.0001 with lognormal Welch’s t-test

To quantify how ICP0 impacts TDP-43 aggregation, we transduced cells with the GFP-TDP43 lentivirus and infected them with wild-type HSV-1 KOS, an ICP0-null virus (7134), or its repaired virus (7134R) (Cai and Schaffer 1989). In accordance with earlier literature, infection with ICP0 competent viruses KOS and 7134R fully disrupted PML bodies (Everett and Maul 1994; Everett et al. 1998; Rodríguez et al. 2020), whereas infection with 7134 left PML bodies mostly intact (Fig. 5a). By contrast, infection with HSV-1 KOS and 7134R led to the formation of TDP-43 nuclear assemblies, whereas infection with 7134 did not (Fig. 5a). Consequently, we observed a significant increase in TDP-43 assembly volume in 7134R infected cells compared to 7134 infected cells [geometric mean volume of 1.435 µm^3^ vs. 0.9048 µm^3^ respectively] (Fig. 5b), an increase similar in scale to that observed between HSV-1 and Mock infected cells [geometric mean volume of 1.416 µm^3^ vs. 0.6486 µm^3^ respectively] (Figs. 2d and 5b). Together, these data show that ICP0 expression is necessary for the formation of nuclear TDP-43 assemblies in HSV-1 infected cells.

### Exogenous ICP0 expression is sufficient to form TDP-43 assemblies

Having identified that ICP0 expression is required for the formation of intranuclear TDP-43 assemblies during HSV-1 infection, we next asked if ICP0 alone was sufficient to induce assembly of TDP-43 in uninfected cells. We generated a lentiviral plasmid that expresses a codon-optimized ICP0 under a doxycycline-inducible promoter (pLVX-ICP0). We transduced ARPE-19 cells for 2 days, after which we treated cells with doxycycline for 9 h and confirmed expression of ICP0 in doxycycline-treated cells by immunoblot (Fig. 5c). Induced expression of ICP0 did not detectably alter TDP-43 levels (Fig. 5c). Using indirect immunofluorescence microscopy, we showed that the ectopically expressed ICP0 localizes to the nucleus in and induces the formation of nuclear TDP-43 assemblies (Fig. 5d). Similar to HSV-1 infection, these assemblies were significantly larger than homeostatic TDP-43 assemblies [geometric mean volume of 0.8988 µm^3^ and 0.3036 µm^3^ respectively] (Fig. 5e). Together, these experiments show that ICP0 is both necessary and sufficient to induce formation of TDP-43 assemblies in the host cell nucleus.

## Discussion

TDP-43 is a critical neuronal disease factor showing proteotoxic cytoplasmic accumulation in motor neurons of individuals with amyotrophic lateral sclerosis. Despite decades of work, physiological triggers leading to aberrant localization and accumulation remain elusive. Here, we showed that infection with the ubiquitous neurotrophic pathogen HSV-1 leads to the formation of TDP-43 assemblies in the nucleus. Using chemical perturbations to infection and genetic virus mutants, we narrowed the formation of nuclear assemblies down to a single viral protein, ICP0. We confirmed that expression of ICP0 is both necessary and sufficient to induce the formation of nuclear assemblies, and that similar assemblies form in both epithelial cells and differentiated neuron-like cells. Together, our data suggests a possible link between a common viral infection and a devastating neurodegenerative disease.

Several cellular stressors alter TDP-43 localization and aggregation, and virus infections provide a powerful experimental tool to study host stress responses. Despite this, the effects of virus infection on TDP-43 binding, expression and localization remain understudied. Here, we show that infection with an α-herpesvirus leads to aberrant nuclear assembly of TDP-43, but other viruses have also been reported to alter TDP-43 localization through distinct mechanisms. Enterovirus encoded proteases have been shown to cleave TDP-43 into shorter, aggregate-prone isoforms leading to the formation of TDP-43 inclusion bodies in vivo (Fung et al. 2015; Xue et al. 2018; Aguglia et al. 2023). Similarly, a viral protein in Theiler’s murine encephalomyelitis virus induces the formation of cytoplasmic TDP-43 aggregates during infection (Masaki et al. 2019). Many RNA viruses activate the integrated stress responses and thereby potently induce stress granule formation, a subcellular structure that is thought to have a transitional role in TDP-43 aggregation (Gasset-Rosa et al. 2019; Mann et al. 2019). However, TDP-43 aggregation has not been systematically examined in the context of viral infection, and a recent study suggests that TDP-43 is not a significant component of virus-induced stress granules (Lang et al. 2026). Interestingly, overexpression of the Kaposi’s sarcoma herpesvirus ORF57 protein was shown to reduce sodium arsenite-induced TDP-43 aggregates (Webber et al. 2023). Lastly, during preparation of this manuscript, a recent preprint reported similar TDP-43 assemblies upon HSV-1 infection in in vitro and in vivo systems, though they did not distinguish these assemblies from anisosomes or tested the necessity of ICP0 and other viral proteins in the context of virus infection (Freisem et al. 2026). Another study reported TDP-43 as an ICP0-dependent ubiquitination target in primary rat neurons (Harrell et al. 2023), indicating a functional interaction between ICP0 and TDP-43. These studies together suggest that viral infections can modulate TDP-43 localization and aggregation, highlighting viruses as both triggers and tools to further elucidate TDP-43 related cellular stress responses in neurodegenerative disease.

ICP0 is canonically understood to be among the first virus-encoded proteins to be expressed during *de novo* HSV-1 infection and has a plethora of roles to prime the host cell for viral infection (Boutell and Everett 2013; Rodríguez et al. 2020). Through its RING finger domain, it ubiquitinates host proteins and marks the majority for degradation by the proteasome, thereby countering innate immune defenses. The RING finger domain of ICP0 is highly conserved across members of the α-herpesvirus family (Rodríguez et al. 2020), and ubiquitination of key targets like PML and IFI16 is observed for some orthologues (Everett et al. 2010; Wang et al. 2011; Girault et al. 2025). It would therefore be interesting to expand further studies of intracellular TDP-43 localization into other α-herpesviruses, particularly VZV. Recent population-based studies demonstrate that VZV vaccination is associated with a delayed onset of neurodegenerative disorders (Taquet et al. 2024; Eyting et al. 2025). Together, these observations raise the possibility that a viral gene function conserved across α-herpesviruses may contribute to the development of neurodegenerative disease.

Cytoplasmic TDP-43 aggregates are considered a hallmark of ALS, however, upon HSV-1 infection we do not detect cytoplasmic accumulation. Instead, we found that TDP-43 forms nuclear assemblies in response to ICP0 expression. Although nuclear TDP-43 aggregates have been reported, they are less studied than their cytoplasmic counterparts. Under homeostatic conditions, TDP-43 undergoes liquid-liquid phase separation (LLPS) into dynamic nuclear droplets with distinct functions (Gasset-Rosa et al. 2019; Pérez-Berlanga et al. 2023; Scherer et al. 2024). The ability of TDP-43 to phase separate is thought to be critical for neuronal homeostasis, and several ALS-linked mutations interfere with this property and thereby promote disease (Johnson et al. 2009; Guenther et al. 2018). Consistent with a functional role for LLPS, one study showed that TDP-43 dynamically colocalizes with paraspeckles through LLPS in response to cellular stress, and that these structures prevent neurotoxicity (Wang et al. 2020). In contrast, other stress-induced nuclear TDP-43 assemblies have been reported to lead to impaired splicing of key neuronal genes such as STMN2 (Huang et al. 2024). It remains to be determined what the functional consequences of the assemblies described in this study are, and whether they might similarly affect TDP-43 nuclear functions.

Of note, although TDP-43 localization is affected by ICP0 expression, we previously showed that in neurons, TDP-43 is uniquely required for ICP0 mRNA splicing and thus viral replication (Braspenning et al. 2025). This functional interdependence between ICP0 and TDP-43 underscores the intricate balance herpesviruses have established with their hosts over millions of years of co-evolution. It also raises the question of whether ICP0-induced assembly of TDP-43 might affect virus replication by altering viral mRNA splicing and processing.

It has been reported that TDP-43 can be sequestered into anisosomes, a shell-like nuclear structure of TDP43 surrounding the molecular chaperone HSP70 (Yu et al. 2021). This requires TDP-43 acetylation and is independent of its RNA-binding function. The lack of spatial overlap in our data suggests these structures are not formed in response to HSV-1 infection. It is notable that herpesvirus infection does induce elevated expression of HSP70 and other chaperones (Santomenna and Colberg-Poley 1990; Kobayashi et al. 1994; Ohgitani et al. 1998), and promotes the formation of nuclear inclusion bodies containing misfolded proteins in an ICP22-dependent manner (Burch and Weller 2004; Livingston et al. 2009; Metzger et al. 2025). However, we found that nuclear TDP-43 assemblies formed in the absence of ICP22, arguing that these virus-induced structures are not equivalent to bona-fide VICE domains. While the exact composition of HSV-1-induced TDP-43 nuclear assemblies remains to be determined, our data suggest they are distinct from chaperone-containing nuclear bodies. Moreover, whether these assemblies are protective or contribute to cytotoxicity remains an open question.

## Materials and methods

### Cells and viruses

ARPE-19 cells were maintained in DMEM: F12 (Gibco) supplemented with 10% heat-inactivated FBS (HI-FBS), penicillin-streptomycin (P/S), 15 mM HEPES and 1 mM sodium pyruvate (Gibco). 293T Lenti-X cells (Takara Bio) were cultured in DMEM with 10% HI-FBS and P/S. Complementing Vero cell lines E5 (for HSV-1 n12 - ΔICP4), V529 (for HSV-1 d301 - ΔICP8), and V22 (for HSV-1 d22lacZ - ΔICP22) were maintained in DMEM (Gibco) + 5% HI-FBS + 1% P/S. Vero and U2OS cells were cultured in DMEM + 5% calf serum + 1% P/S. HSV-1 KOS and 7134R were propagated and titered on Vero cells, and ICP0-mutant virus (7134) on U2OS cells. Mutant viruses and complementing cell lines used in this study were kindly donated from the following labs: 7134 (ΔICP0), 7134R and n12 (ΔICP4) by Neal DeLuca, University of Pittsburgh, d22lacZ (ΔICP22) by Steven Rice, University of Minnesota, and d301 (ΔICP8) by David Knipe, Harvard Medical School.

HD10.6 cells were provided by Anna Cliffe, University of Virginia and were propagated in proliferation media and differentiated using maturation media to neuron-like cells as described (Thellman et al. 2017; Braspenning et al. 2025). All cells were maintained at 37 °C and 5% CO_2_ in a humidified incubator.

### Cloning and lentivirus generation

To generate an inducible ICP0 vector, the ICP0 coding sequence (derived from NC_001806.2) was synthesized as codon-optimized gBlock (IDT Technologies) to reduce the internal repetitiveness and GC-content of the open reading frame and was subcloned into the pLVX-Tet-One Puro lentiviral vector using the EcoRI and BamHI sites to generate pLVX-ICP0. For overexpression of the GFP-TDP43 fusion protein, pHBS1147 (Addgene plasmid #118797, (Schmidt et al. 2019) was used as a lentiviral transfer plasmid. Lentivirus were generated as described before on 293T Lenti-X cells (Braspenning et al. 2025).

### Transduction and doxycycline induction

For transduction, 30 µL lentivirus was used per well of a 24-wells plate and reverse transduction was performed in the presence of 8 µg/mL polybrene. Infectious media was replaced for culture media the next day, and cells were used for assays at 3 days post-transduction. To induce expression of ICP0, cells were treated with 3 µg/mL of doxycycline (Sigma) for 9 h.

### Immunoblotting

Cells were lysed using 1x Laemmli buffer, followed by incubation at 95℃ for 10 min. Proteins were then separated by SDS-polyacrylamide gel electrophoresis and transferred onto nitrocellulose membranes using a wet transfer. Non-fat dry milk (5% in TBS-T) was used to block the membranes, followed by overnight incubation at 4℃ with primary antibodies diluted in TBS-T + 3% BSA + 0.02% NaN_3_. The primary antibodies used were: rabbit anti-TDP43 (10782-2-AP, Proteintech), rabbit anti-GAPDH (2118, Cell Signaling Technologies), mouse anti-ICP0 (ab6513, Abcam), mouse anti-ICP4 (ab6514, Abcam) and rabbit anti-GFP (2956, Cell Signaling Technologies). The next day, membranes were washed thrice with TBS-T and incubated with HRP-conjugated secondary antibodies (Cytiva) in TBS-T + 5% milk. Excess antibody was removed by washing with TBS-T, and chemiluminescent signal was visualized on an iBright CL1000 imaging system using the Pierce ECL Western Blotting kits (ThermoFisher Scientific).

### Immunofluorescence

Cells were plated (ARPE-19) or differentiated (HD10.6) on glass coverslips prior to usage, infected as indicated and fixed for 15 min using 4% paraformaldehyde. Next, cells were permeabilized using 0.1% Triton X-100 in PBS for 10 min and blocked with 5% normal goat or 4% human serum diluted in PBS. Coverslips were then incubated with primary antibodies overnight at 4℃ or for 1 h at room temperature in PBS. Primary antibodies used for immunofluorescence include: mouse anti-TDP43 (MAB7778, R&D Systems), rabbit anti-TDP43 (10782-2-AP, Proteintech), chicken anti-VP22 (Aves labs), mouse anti-ICP4 (ab6514, Abcam), mouse anti-ICP0 (ab6513, Abcam), rabbit anti-HSP70 (10995-1-AP, ProteinTech), anti-PML (sc5621, Santa Cruz), Next, coverslips were washed and incubated for 1 h at room temperature with secondary antibodies (AF-488, AF-555 or AF-694 conjugated goat antibodies, all ThermoFisher) in PBS. Finally, the coverslips were washed, nuclei were counterstained with Hoechst 33,342 (Life Technologies) or DAPI (D1306, Invitrogen) in PBS for 5 min and mounted using Fluorshield mounting media (Sigma). Imaging acquisition was performed using a Keyence BZ-X800 imager, a Zeiss LSM800 confocal microscope or a Nikon Ti2 × 1 spinning disc confocal microscope as described below. Images were adjusted for brightness and contrast using open-source Fiji software or Adobe Photoshop. Scale bars represent 10 μm.

### High-content imaging and analysis

After staining as described above, the Keyence BZ-X800 imager was used for high-content image analysis. In brief, the 20x lens was used to capture images at random locations in the coverslips or the well in the blue channel (Hoechst/DAPI) and green channel (GFP).

Analysis was performed single-blinded using Fiji software. In brief, the number of nuclei per image were determined using the *Analyze Particles* tool, and the outlines were stored as Regions Of Interests (ROI). In each ROI, the GFP-signal intensity was measured to determine the number of transduced (GFP+) cells. Finally, the quantity of large GFP-TDP43 assemblies per image was determined by using the *Find Maxima* tool with a noise tolerance of 75 and divided by the amount of GFP+ cells to determine the average amount of large assemblies per cell.

### Confocal imaging and analysis

For confocal immunofluorescence imaging experiments, cells were mounted on a Nikon Ti2 × 1 spinning disc confocal microscope and images were captured using a Prime 95B scMOS camera (Photometrics) with a 60x/1.49 numerical aperture objective lens with DIC capability. Fluorescence signals were obtained using 405 nm, 488 nm, 561 nm, and 640 nm lasers with the following emission filters: 455/50, 525/36, 605/52, and 705/72 (Chroma Technology Corp). Z-stack images were collected with 0.5 μm spacing for a total depth of 12–20 μm.

Analysis of condensate Z-stack images was performed single-blinded using detect-condensates, a python analysis pipeline we are developing in house (https://github.com/liamholtlab/foci_counting/blob/main/3d_detection/detect_condensates.ipynb*).* Within the detect-condensates pipeline, we used the cellpose python package (https://github.com/mouseland/cellpose, (Stringer et al. 2021) to generate regions of interest (ROI) for marking individual cells based on the nuclear DAPI stain. Outputs from detect-condensates were analyzed using a jupyter notebook in VS Code to determine the volume of each condensate.

To determine fluorescent intensity profiles across individual assemblies, images were taken using a Zeiss LSM800 confocal microscope with 63x objective. Lines of roughly equal length were drawn in Fiji, and multichannel intensity profiles were generated using the “multichannel plot profile” macro provided by Michael Cammer (Microscopy Core, NYU Langone Health). Reported intensities were then normalized using a custom Python script. For each assembly the center was defined by the peak GFP-TDP43 signal, and distances were normalized relative to this center. Normalized profiles were then binned along the distance axis and averaged across assemblies. From this, intensity profiles were plotted with a 95% confidence interval, using a moving average for visualization purposes.

### Statistical analysis

Figures show individual data points or mean +/- SEM. All statistical analysis were done with Graphpad Prism 10 software. * = *p* < 0.05, ** = *p* < 0.01, *** = *p* < 0.001, **** = *p* < 0.0001 according to the statistical test indicated in the figure legends. If no statistics are given, the difference between conditions was not significant.

## Data Availability

No datasets were generated or analysed during the current study.
